# Integrated Multi-Omics Analysis Reveals the Effect of Maternal Gestational Diabetes on Fetal Mouse Hippocampi

**DOI:** 10.3389/fcell.2022.748862

**Published:** 2022-02-14

**Authors:** Si-si Luo, Ke-xin Zou, Hong Zhu, Yi Cheng, Yi-shang Yan, Jian-zhong Sheng, He-feng Huang, Guo-lian Ding

**Affiliations:** ^1^ The International Peace Maternity and Child Health Hospital, School of Medicine, Shanghai Jiao Tong University, Shanghai, China; ^2^ Shanghai Key Laboratory of Embryo Original Diseases, Shanghai, China; ^3^ Obstetrics and Gynecology Hospital, Institute of Reproduction and Development, Fudan University, Shanghai, China; ^4^ Research Units of Embryo Original Diseases, Chinese Academy of Medical Sciences, Shanghai, China; ^5^ The Key Laboratory of Reproductive Genetics (Zhejiang University), Ministry of Education, Hangzhou, China

**Keywords:** gestational diabetes mellitus, fetal mouse brain, hippocampus, metabolomics, DNA methylation, transcriptomics

## Abstract

Growing evidence suggests that adverse intrauterine environments could affect the long-term health of offspring. Recent evidence indicates that gestational diabetes mellitus (GDM) is associated with neurocognitive changes in offspring. However, the mechanism remains unclear. Using a GDM mouse model, we collected hippocampi, the structure critical to cognitive processes, for electron microscopy, methylome and transcriptome analyses. Reduced representation bisulfite sequencing (RRBS) and RNA-seq in the GDM fetal hippocampi showed altered methylated modification and differentially expressed genes enriched in common pathways involved in neural synapse organization and signal transmission. We further collected fetal mice brains for metabolome analysis and found that in GDM fetal brains, the metabolites displayed significant changes, in addition to directly inducing cognitive dysfunction, some of which are important to methylation status such as betaine, fumaric acid, L-methionine, succinic acid, 5-methyltetrahydrofolic acid, and S-adenosylmethionine (SAM). These results suggest that GDM affects metabolites in fetal mice brains and further affects hippocampal DNA methylation and gene regulation involved in cognition, which is a potential mechanism for the adverse neurocognitive effects of GDM in offspring.

## Introduction

Accumulating human and animal studies have revealed that the early developmental environment is closely related to the long-term health of offspring (Hanson and Gluckman, 2014). Gestational diabetes mellitus (GDM), with hyperglycemia *in utero* during pregnancy, has been increasing in last decade, surging from 2 to 6% in 2009 to 12–18% in 2020 (Alwan et al., 2009; Chiefari et al., 2017; Dalfrà et al., 2020). Previous studies have demonstrated that perinatal hyperglycemia could induce abnormal glucose metabolism in offspring (Ding et al., 2012; Chandna et al., 2015; Zhu and Zhang, 2016). Notably, maternal diabetes not only cause abnormal glucose tolerance and insulin secretion but also affects the development of the nervous system in offspring (Valle-Bautista et al., 2020; He et al., 2021). Both human cohort studies and experimental studies using rodent models have linked GDM with impaired cognitive abilities and decreased learning and memory in offspring (Kinney et al., 2003; Fraser et al., 2014; Bytoft et al., 2016; Daraki et al., 2017; He et al., 2017; de Sousa et al., 2019). However, the mechanisms that link GDM to the development of cognitive impairments in offspring have not been elucidated.

Intrauterine exposure to adverse environments may disrupt the normal development pattern of the fetus in the structure, physiology, and metabolism. Brain development is particularly sensitive to environmental influence during the early life stage (Lindsay et al., 2019). During the period of maternal GDM, the fetal brain was directly exposed to an intrauterine hyperglycemic environment before birth. Therefore, the offspring of GDM may also suffer from changes in brain function, behavior, and even the development of brain diseases (Aviel-Shekler et al., 2020). As a structure critical to cognitive processes, the hippocampus of the brain has been shown to undergo apoptotic cell death when subjected to hyperglycemic insult (Hami et al., 2013; Kuang et al., 2014; Haghir et al., 2017). Diabetes during pregnancy also influences the expression of genes associated with hippocampal development (Hami et al., 2013). However, the underlying mechanism of GDM-induced abnormal gene expression and regulation of the hippocampus remains obscure.

Epigenetic modifications such as DNA methylation provide a possible link between intrauterine hyperglycemia exposure early in development and cognitive disorders later in life. The status of DNA methylation is very sensitive to cell metabolism and nutritional status (Li et al., 2018; Muehlmann et al., 2020). Emerging studies have shown that metabolism influences the genomic status by regulating the enzymes for DNA methylation (Martínez-Reyes and Chandel, 2020). One carbon metabolism, composed of the folate and methionine cycle, generates universal methyl donor S-adenosyl-l-methionine (SAM), which is a substrate for DNA methyltransferases (DNMTs) (Mentch and Locasale, 2016). The tricarboxylic acid (TCA) generates metabolites that link energy pathways with epigenetic modifications. Alpha-ketoglutarate (α-KG) is a substrate for TET enzymes, flavin adenine dinucleotide (FAD) is an essential cofactor for the lysine demethylases LSD1 and LSD2 (Bartke and Schneider, 2020). Beside, succinic acid and fumaric acid can inhibit function of DNA demethylase–TETs (Martínez-Reyes and Chandel, 2020).

One of the most widely used drugs for diabetic animal model is streptozotocin (STZ), a chemical drug specifically inducing damage of β-cell of islet (Pasek and Gannon, 2013). In our present study, we established the mouse model of GDM by STZ injection of pregnant mice, which has been used in our previous studies (Ding et al., 2012; Zhu et al., 2019; Zou et al., 2021). We analyzed the ultrastructure of the hippocampus by electron microscopy. Then, we profiled the methylome and transcriptome of fetal hippocampi simultaneously to identify the key factors that were responsible for fetal cognitive dysfunction exposed to an intrauterine hyperglycemic environment. We further used a mouse model that mimicked clinical GDM and profiled the metabolome of fetal brains to explore the potential biochemicals and pathways involved in DNA methylation.

## Materials and Methods

### Animals and Experimental Design

All animal protocols were approved by the Zhejiang University Animal Care and Use Committee. All experiments were performed with Institute of Cancer Research (ICR) mice. Eight-week-old virgin ICR females (*n* = 40) were mated with normal ICR males. Pregnancy was dated by the presence of a vaginal plug (designated day 0 [D0] of pregnancy). After a 12-h fast, the D0 females were randomly divided into a control group (Ctrl) and a GDM group. Mice in the GDM group were injected with a single intraperitoneal injection of streptozotocin (STZ; Sigma, St. Louis, MO) in 0.1 mmol/L citrate buffer (pH 4.5) at a dose of 150 mg/kg body weight. Control pregnant mice received an equal volume of citrate buffer. Blood glucose levels were measured at day 3, day 7 and day 18.5 of pregnancy via the tail vein, and diabetes was defined as a blood glucose level between 14 and 19 mmol/L as previously described (Ding et al., 2012). At embryonic day 18.5 (E18.5), the fetal mice were removed from 9 dams per group by abdominal cesarean section after immediate euthanization. Then, the meninges were carefully removed, and the hippocampi were dissected for electron microscopy (*n* = 4 per group) and analysis of the methylome and transcriptome (*n* = 3 per group) (Supplementary Figures S1A,C,D).

To further mimic the hyperglycemic environment in the middle and late trimesters of most clinical GDM, on day 6 and day 12 of pregnancy, females fasting for 8 h received an intraperitoneal injection of STZ as the late GDM group (*n* = 15). Pregnant females in the Ctrl group (*n* = 15) received an equal volume of citrate buffer. Blood glucose levels were measured via the tail vein, and diabetes was defined as a blood glucose level between 14 and 19 mmol/L (Zhu et al., 2019). At E18.5, the fetal mice brains were isolated from the dams by abdominal cesarean section after immediate euthanization. Then, untargeted metabolomics was performed on the whole brains of E18.5 fetal mice from Ctrl group (*n* = 7) and GDM group (*n* = 10) (Supplementary Figures S1B,D).

### Electron Microscopy

Samples (*n* = 4 per group) were fixed in 2.5% glutaraldehyde and postfixed with 3% osmium tetroxide (OsO4) for 2 h. The samples were dehydrated in a graded series of ethanol, embedded in Epon resin, sliced into 100 nm pieces and finally imaged with a transmission electron microscope at 80 kV (Hitachi, Japan, H7650 TEM).

### Reduced Representation Bisulfite Sequencing (RRBS)

Reduced representation bisulfite sequencing (RRBS) was performed in hippocampi obtained at embryonic day 18.5 from the Ctrl and GDM group (*n* = 3 per group) (Genergy Biotechnology Co., Ltd., Shanghai, China). Briefly, 5 μg genomic DNA was digested using the methylation-insensitive restriction enzyme MspI (New England Biolabs, Beverly, MA, United States). A Qiagen Mini Purification kit (Qiagen, Hilden, Germany) was used to purify the digested products. Then, the ends of each restriction fragment were filled in, and adenosine was added at the 3′-end. Methylated paired-end Illumina adapters were ligated to the ends of the DNA fragments using T4 DNA ligase, and fragments sized 100–200 bp were purified by agarose gel extraction. The purified fragments were treated with sodium bisulfite and then amplified by PCR. The final PCR products were sequenced on HiSeq 3,000 (Illumina Inc., San Diego, CA, United States).

### RNA Isolation and RNA-Seq Analysis

Hippocampi from the Ctrl and GDM groups (*n* = 3 per group) were isolated from fetal mice and snap frozen in liquid nitrogen (for RNA-seq). Total RNA was isolated from hippocampi using Trizol. The purity of RNA was determined using a NanoDrop (Thermo, United States) spectrophotometer (OD260/280 is 1.8–2.2), and the integrity of RNA was assessed by electrophoresis (Tianneng, China) using agarose gel (28S:18S > 1.5). mRNA was enriched by magnetic beads with oligo (dT) and fragmented into 200 bp. RNA-seq libraries underwent 15 cycles of PCR amplification to obtain cDNA library. After RNA library construction, Qubit (Invitrogen, United States) was used to detect accurate RNA concentrations (≥500 ng/μl). Paired-end sequencing with a read length of 151 bp was conducted using the Illumina HiSeq3000 (Illumina Inc., San Diego, CA, United States) platform.

### Ultraperformance Liquid Chromatography-Mass Spectrometry (UPLC-MS)

100 mg (±2%) of each sample was weighed and transferred into a 5 ml EP tube, which was prepared with 2 ml tissue extract (75% 9:1 methanol: chloroform, 25% ddH2O) (−20°C) and 3 steel beads, and then grinded for 60 s at 50 Hz in a high-throughput tissue grinder twice. Ultrasound-assisted extraction was processed at room temperature for 30 min and then the samples were put on ice for 30 min. After that, samples were centrifuged at 14,000 rpm for 20 min at 4°C and 1250 μl of the supernatant from each sample were transferred into another 2 ml centrifuge tube. Then, samples were concentrated to dry in vacuum and dissolved with 200 μl 2-chlorobenzalanine (4 ppm) 50% acetonitrile solution and the supernatant was filtered through 0.22 µm membrane to obtain the prepared samples for LC-MS. Every 20 ul of each sample was mixed as a quality control (QC) sample. The rest of the samples were used for LC-MS detection.

Chromatographic separation was accomplished in a Thermo Ultimate 3,000 system equipped with an ACQUITY UPLC^®^ HSS T3 (150 × 2.1 mm, 1.8 μm, Waters) column maintained at 40°C. The temperature of the autosampler was 8°C. Gradient elution of analytes was carried out with 0.1% formic acid in water (C) and 0.1% formic acid in acetonitrile (D) or 5 mM ammonium formate in water (A) and acetonitrile (B) at a flow rate of 0.25 ml/min. Injection of 2 μl of each sample was performed after equilibration. An increasing linear gradient of solvent B (v/v) was used as follows: 0–1 min, 2% B/D; 1–9 min, 2–50% B/D; 9–12 min, 50–98% B/D; 12–13.5 min, 98% B/D; 13.5–14 min, 98–2% B/D; 14–20 min, 2% D-positive model (14–17 min, 2% B-negative model).

The ESI-MSn experiments were executed on a Thermo Q Exactive mass spectrometer with spray voltages of 3.8 kV and −2.5 kV in positive and negative modes, respectively. Sheath gas and auxiliary gas were set at 30 and 10 arbitrary units, respectively. The capillary temperature was 325°C. The analyzer scanned over a mass range of m/z 81–1,000 for full scan at a mass resolution of 70,000. Data-dependent acquisition (DDA) MS/MS experiments were performed with HCD scans. The normalized collision energy was 30 eV. Dynamic exclusion was implemented to remove some unnecessary information in MS/MS spectra.

### Data Analysis

RRBS data were analyzed using the R package *minfi* for raw data preprocessing (quality control and normalization). RRBS reads were mapped to the reference mouse genome (mm10) by Bismark (version 0.16.3). DSS V2.30.1 in R was used to detect DMLs. Differentially methylated loci (DMLs) were analyzed based on a Bayesian approach (Feng et al., 2014) and were summarized as follows: two groups were modeled according to the Bayesian stratification model, and the Wald test was applied to each locus to obtain a *p*-value for each CpG site. For each CpG site, a difference in methylation value between two groups ≥5% and a posteriori probability of Wald test ≥0.95, or *p*-value < .01 was considered to be a differentially methylated loci (DML). A methylation region was defined as a differently methylated regions (DMR) when it met the following three criteria: 1) the length of this region was at least 50 bp; 2) the region contained no less than three CpG sites; and 3) the proportion of DMLs in this region was no less than 50%. When a DMR showed no less than 50% overlap with one element of the gene, it was defined as a differentially methylated gene (DMG).

Quality control of the raw and trimmed reads of RNA-seq was performed using FastQC (v0.11.5). The STAR (2.5.3a) software package performed the task that mapped high-throughput sequencing reads to a reference genome in RNA-seq data analysis (Dobin et al., 2013). The DEseq2 package v1.16.1 in R was used to identify the differentially expressed genes (DEGs) in GDM hippocampi compared with the Ctrl group (Love et al., 2014). The *t*-test and Benjamini–Hochberg method were used to calculate the *p*-value and FDR, respectively. By using a stringent threshold and significant criteria of FDR <0.05 and >1.2-fold change or <0.833-fold change, a total of 953 DEGs were screened out.

Metabolites were identified according to their exact molecular weight and the MS/MS fragmentation pattern by a comparison with those in the online Metlin database (http://metlin.scripps.edu), MoNA (https://mona.fiehnlab.ucdavis.edu//). The mass error was 15 ppm. Score plots of orthogonal partial least-squares discriminant analysis (OPLS-DA) clearly separated the GDM group from the control group. The variable importance in projection (VIP) generated in OPLS-DA processing represented the contribution to the discrimination of each metabolite ion between groups. The *t*-test was used to calculate the *p*-value. The significantly differential metabolites were identified using the threshold of *p*-value < .05 and VIP ≥1. MetaboAnalyst 4.0 (www.metaboanalyst.ca) was used for metabolic pathway analysis based on the Kyoto Encyclopedia of Genes and Genomes (KEGG) database. Enrichment for each identified pathway was calculated using Fisher’s exact test and *p*-value < .05 was considered statistically significant.

### Pathway Analysis

ClusterProfiler V3.8.0 in R was used to identify and visualize the gene ontology (GO) terms and KEGG pathways enriched by the DMGs and shared genes of DEGs and DMGs. To identify the function of the aforementioned DEGs, KEGG analyses were performed by Cytoscape ClueGO (v2.5.7) and CluePedia (v1.5.7). Gene enrichment for each identified pathway was calculated using Fisher’s exact test and *p*-value was set to .05.

### Functional Network Analysis

To screen for functional networks among the differentially methylated and expressed genes, we employed the Functional Networks of Tissues in Mouse (FNTM) prediction tool for mouse tissue-specific protein interactions, which integrates genomic data and prior knowledge of gene function (Goya et al., 2015). We used the hippocampus tissue database and only kept edges with a relationship confidence greater than 0.75.

An interactome network model integrating transcriptomics and metabolomics was constructed with Cytoscape MetScape (v3.1.3).

### Statistical Analysis

Statistical analyses of results of metabolites content were performed with GraphPad Prism (version 8.0). Statistical significance was determined by two-tailed unpaired Student’s *t*-test. *p*-value < .05 was considered statistically significant.

## Results

### Abnormal Nuclear Ultrastructure of Neural Cells in GDM Fetal Mice Hippocampi

**FIGURE 1:**
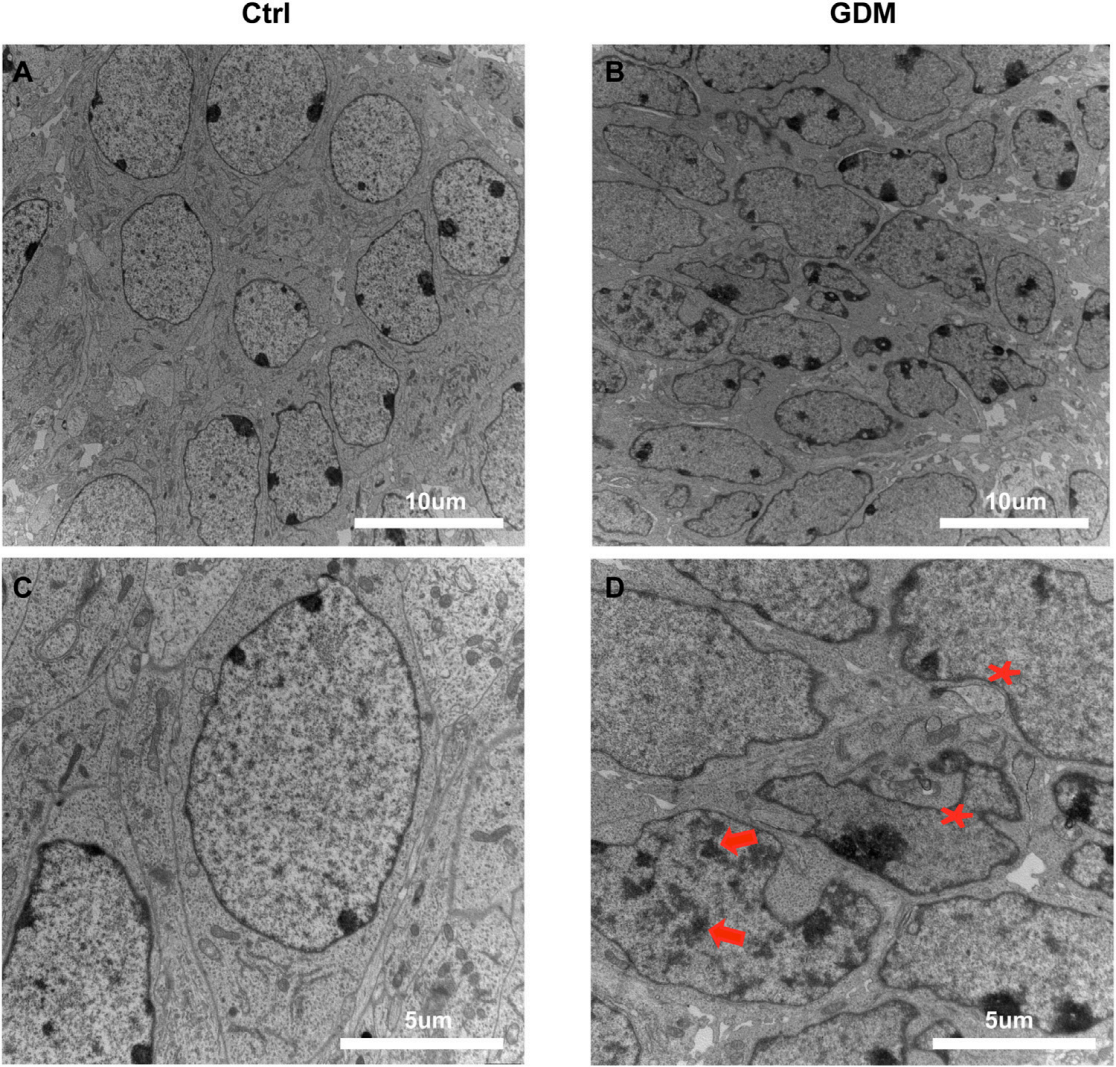
Ultrastructure of the hippocampus in fetal mice using transmission electron microscopy. **(A,C)** Neural cells of the fetal hippocampus via transmission electron microscopy in the Ctrl group. Scale bar, 5 μm **(A)**; Scale bar, 2 μm **(C)**. **(B,D)** Neural cells of the fetal hippocampus *via* transmission electron microscopy in the GDM group. Scale bar, 5 μm **(B)**; Scale bar, 2 μm **(D)**. In GDM group, the chromatin aggregated into several clumps (arrows); the shape of the nucleus was irregular; and the nuclei shrinkage was obvious (asterisk).

We used transmission electron microscopy (TEM) to examine nuclei in the CA1 region of hippocampal neural cells. In a part of neurons, the chromatin aggregated into several clumps (Figure 1D, arrows); the shape of the nucleus was irregular; and the invaginations of nucleolemma became evident indicating the shrinkage of the nuclei (Figure 1D, asterisk), suggesting that the nuclei of neural cells were impaired in the GDM group compared to the Ctrl group (Figure 1).

### DNA Methylation Profile of Fetal Mice Hippocampi was Affected in the GDM Group

**FIGURE 2:**
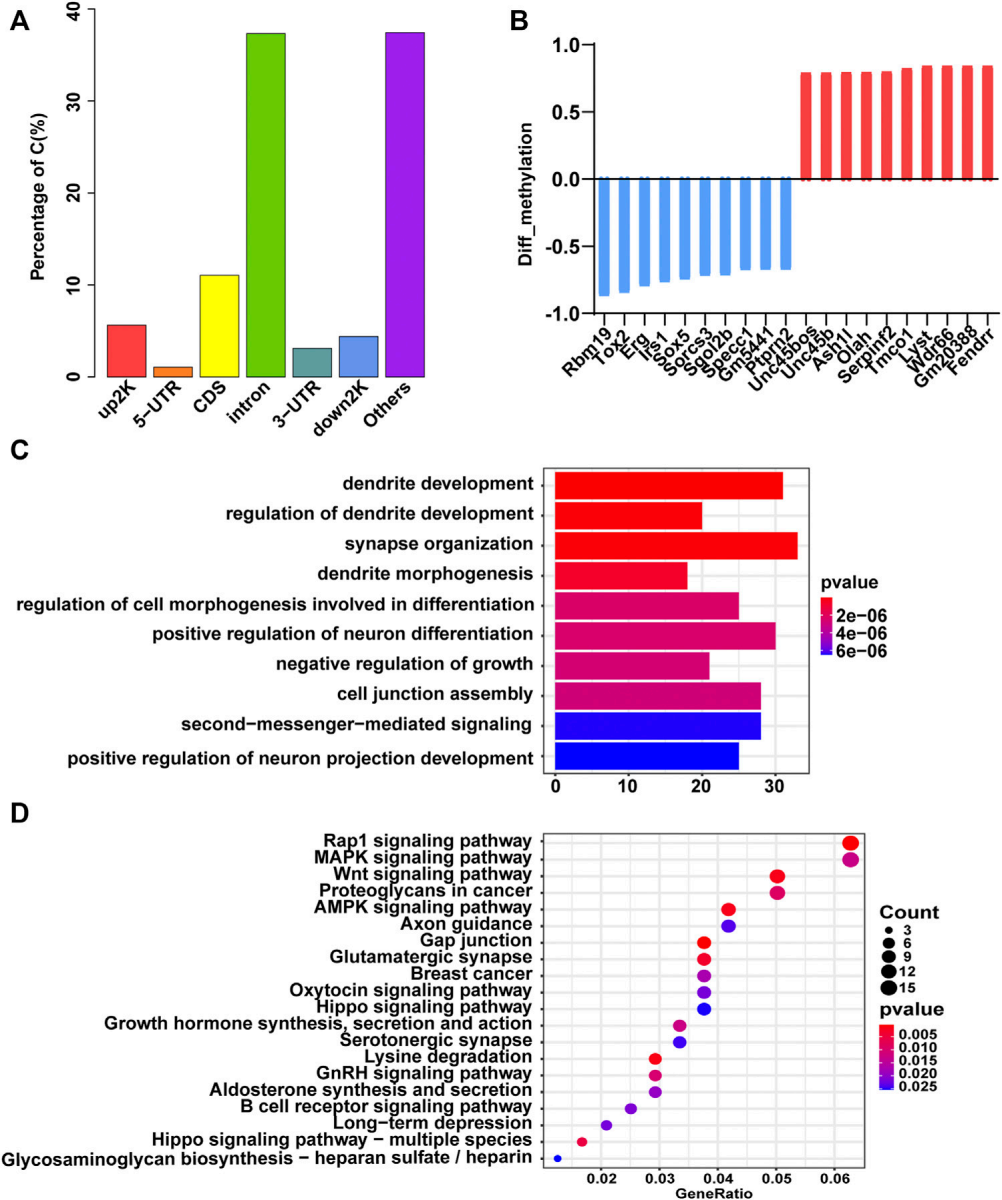
Gene methylation analysis of the GDM and control groups. **(A)** Bar plot of the distribution of differentially methylated loci in different components of the genome. **(B)** The top 10 hypomethylated (blue) and hypermethylated (red) genes. **(C)** GO terms of biological process enriched by DMGs. The color gradient from dark blue to dark red indicates smaller *p*-value. **(D)** Bubble plot of the top 20 Kyoto Encyclopedia of Genes and Genomes (KEGG) enrichment results of DMGs. The size of the circle corresponds gene number enriched in the pathway. The color gradient from dark blue to dark red indicates smaller *p*-value.

We performed RRBS to search for DMRs in GDM group vs. Ctrl group. Initial principal component analysis (PCA) of DMLs revealed a clear distinction between GDM and control samples (Supplementary Figure S2; Supplementary Table S1). The distribution of DMRs in the different functional components of the genome was illustrated in Figure 2A in the upstream 2 k (5.64%), 5′-untranslated region (5′-UTR, 1.06%), coding sequence (CDS, 11.06%), introns (37.31%), 3′-UTR (3.11%), downstream 2 k (4.4%), and other elements (37.42%) of genes. In GDM group, a total of 429 DMR-related genes had lower methylation levels, and 321 DMR-related genes had higher methylation levels (Supplementary Table S2). The top 10 hypomethylated genes (*Rbm19*, *Tox2*, *Erg*, *Irs1*, *Sox5*, *Sorcs3*, *Sgol2b*, *Specc1*, *Gm5441*, *Ptprn2*) and hypermethylated genes (*Unc45bos*, *Unc45b*, *Ash1L*, *Olah*, *Serpinf2*, *Tmco1*, *Lyst*, *Wdr66*, *Gm20388*, *Fendrr*) were shown in Figure 2B. Among them, *Sox5* controls neural differentiation and genesis (Lai et al., 2008). *Sorcs3* is a stronger regulator of glutamate receptor functions compared to GABAergic mechanisms in the hippocampus (Christiansen et al., 2017). The top 10 terms of the GO biological process analysis were shown in Figure 2C, among which “dendrite development,” “regulation of dendrite development,” and “synapse organization” were thought to be important for cognition, learning and memory. In addition, the top 20 pathways of KEGG enrichment were shown in Figure 2D, and some of these enriched terms were involved in “axon guidance,” “glutamatergic synapse,” and “long-term depression,” which were closely related to cognitive function.

### Transcriptome of Fetal Mice Hippocampi was Changed in the GDM Group

**FIGURE 3:**
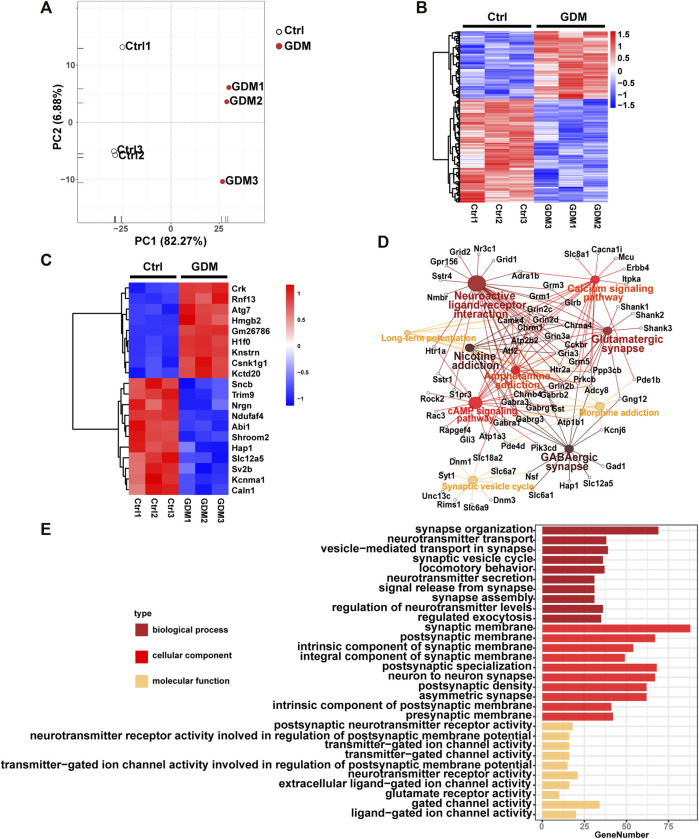
Gene expression analysis of the GDM and control groups. **(A)** Supervised PCA of DEGs. **(B)** Hierarchically clustered heatmap of DEGs. Data are expressed as Z score-normalized values of FPKM. The color gradient from dark blue to dark red in the heatmap indicates increasing transcript levels. **(C)** The top 10 down-regulated and up-regulated genes. Data are expressed as Z score-normalized values of FPKM. The color gradient from dark blue to dark red in the heatmap indicates increasing transcript levels. **(D)** Top 10 Kyoto Encyclopedia of Genes and Genomes (KEGG) pathways of DEGs. The solid circle represents the enriched pathway. The open circle represents genes enriched in the KEGG pathways. A larger size of a solid cycle means more enriched genes. The deeper color of the solid cycle represents a lower *p* value. **(E)** Gene ontology (GO) enrichment of DEGs, including BP (biological process), CC (cellular component), and MF (molecular function).

The gene expression profiles of fetal mice hippocampi at embryonic day 18.5 from the Ctrl and GDM groups were analyzed using RNA-seq. Initial principal component analysis (PCA) revealed a clear distinction between GDM and control samples (Figure 3A). By using a stringent threshold and significant criteria of FDR <0.05 and fold change >1.2, a total of 953 DEGs, including 378 up-regulated and 575 down-regulated genes, were identified in the GDM group compared to the Ctrl group (Figure 3B, Supplementary Table S3). Among the top 20 DEGs, 11 genes (*Ndufaf4*, *Nrgn*, *Hap1*, *Sv2b*, *Slc12a5*, *Sncb*, *Caln1*, *Kcnma1*, *Abi1*, *Trim9*, *Shroom2*) were down-regulated, and 9 genes (*Hmgb2*, *Kctd20*, *H1f0*, *Rnf13*, *Atg7*, *Knstrn*, *Csnk1g1*, *Crk*, *Gm26786*) were up-regulated (Figure 3C). *Nrgn* is expressed exclusively in the brain, particularly in dendritic spines and has been implicated in hippocampal plasticity. *Hap1* depletion impaired postnatal neurogenesis in the dentate gyrus (DG) of the hippocampus (Xiang et al., 2015). *Sv2b* has been shown to be involved in the regulation of synaptic vesicle exocytosis and plays a role in neuronal excitability (Stout et al., 2019). *Caln1* participates in the regulation of trans-synaptic signaling (Reshetnikov et al., 2020).

Functional enrichment analysis identified KEGG pathways including “GABAergic synapse,” “neuroactive ligand-receptor interaction,” “glutamatergic synapse,” “cAMP signaling pathway,” “synaptic vesicle cycle” and “long-term potentiation” related to brain cognition. The top 10 KEGG pathways were illustrated in Figure 3D. In addition, GO analysis revealed that the terms “synapse organization,” “neurotransmitter transport,” “signal release from synapse,” “neuron to neuron synapse” and “postsynaptic neurotransmitter receptor activity” were enriched (Figure 3E).

### Integrated Analysis Identifies Overlapping Genes With Coupled DNA Methylation and Gene Expression

**FIGURE 4:**
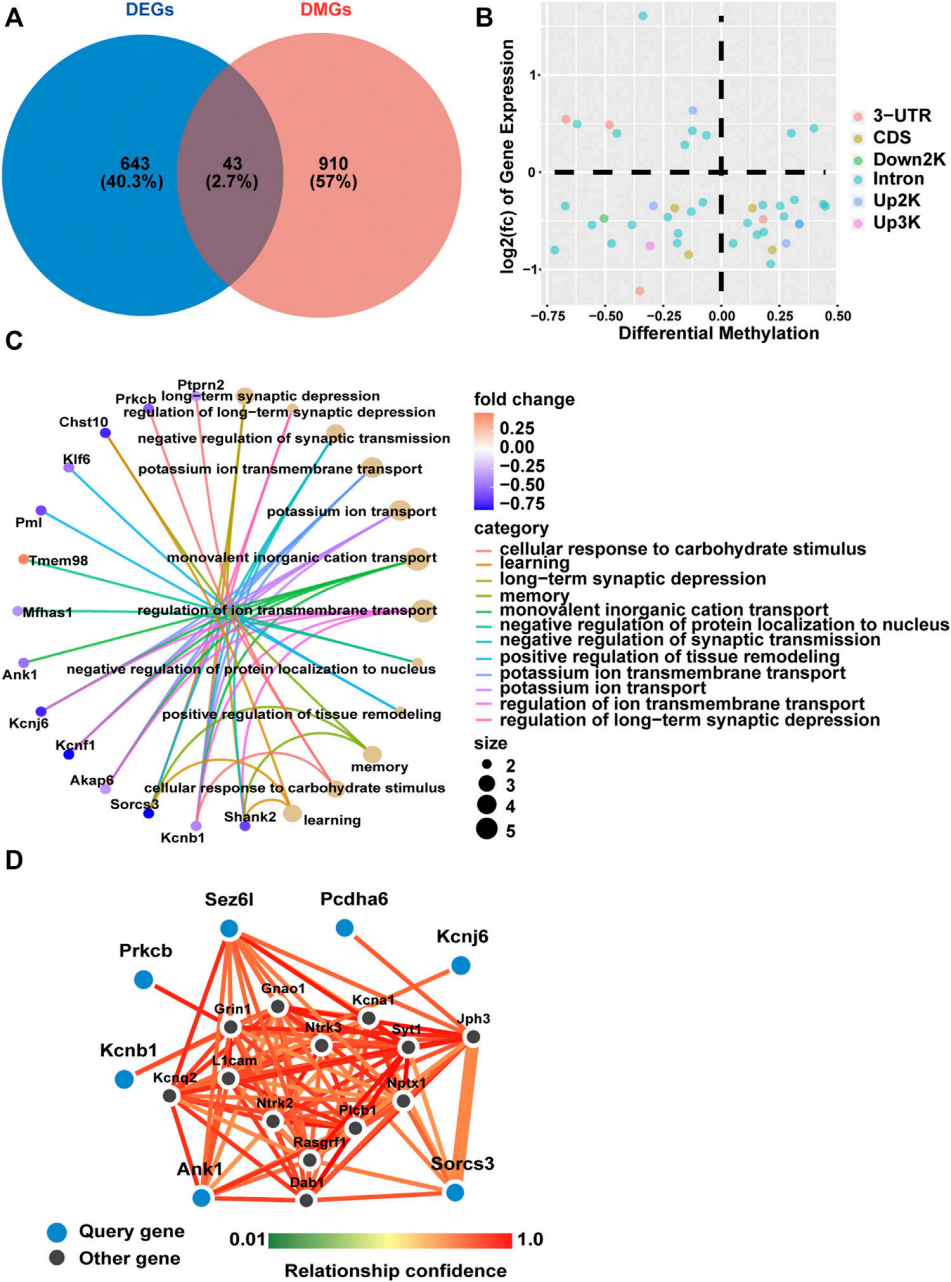
Gene expression and methylation co-analysis. **(A)** Venn diagram illustrating the overlap of differentially expressed genes and differentially methylated genes. **(B)** Relationship of transcriptomic (*y*-axis) and methylation (*x*-axis) profiling based on overlapping genes of DEGs and DMGs. Different colors represent different methylated positions in gene elements. **(C)** Gene ontology (GO) enrichment analysis of biological process (BP) based on shared genes of RNA-seq and RRBS. The color gradient from dark purple to dark pink in the heatmap indicates fold change of DEGs. The bubble size is related to the impact of the pathway. **(D)** Network analysis based on differentially methylated and differentially expressed genes with a relationship confidence greater than 0.75. Larger blue circles indicate candidate genes identified from our analysis, and smaller black circles denote imputed interacting genes in the hippocampus.

We further identified DNA methylation changes linked to gene expression differences in fetal mice hippocampi in the GDM group compared with the Ctrl group. A Venn diagram was constructed to illustrate 43 shared genes of the methylome and transcriptome (Figure 4A). A scatter diagram of the 4-quadrant plots showed that 16 genes (*Cdipt*, *Mfhas1*, *Slc7a14*, *Gm38393*, *Pcdha6*, *Paqr9*, *Rexo2*, *Tbc1d30*, *Akap6*, *Kcnf1*, *Sv2c*, *Arhgap26*, *Ank1*, *Arhgap23*, *Shank2*, *Sez6l*) were identified as hypermethylated and down-regulated, and 9 genes (*Thtpa*, *Sema5b*, *Lima1*, *Gse1*, *Tmem98*, *Mpped2*, *Peg12*, *Mcm10*, *Mid1*) were hypomethylated and up-regulated in the GDM group. The other 18 genes did not show a canonical association of gene expression and methylation status (Figure 4B).

GO analysis showed that the above overlapping genes were mainly enriched in “long-term synaptic depression,” “negative regulation of synaptic transmission” and “learning and memory” (Figure 4C). Functional enrichment analysis identified 17 KEGG pathways that were significantly different between the GDM and Ctrl groups. The top 10 KEGG pathways included “GABAergic synapse,” “glutamatergic synapse,” “serotonergic synapse” and “dopaminergic synapse,” mainly relating to information transfer and cognition (Supplementary Figure S3).

Among all 43 genes that were differentially methylated and expressed, we obtained the seven most functionally connected predicted interactor genes visualized using the FNTM tool (*Pcdha6*, *Ank1*, *Sez6l*, *Kcnb1*, *Kcnj6*, *Prkcb*, *Sorcs3*) (Figure 4D), all of which were down-regulated in transcriptome profiling. These genes are involved in synapse signal transmission, such as *Kcnj6* and *Prkcb*, and function in cognition, such as *Ank1*, *Kcnb1* and *Sorcs3*.

### Altered Metabolite Profiling of GDM Fetal Mice Brains

**FIGURE 5:**
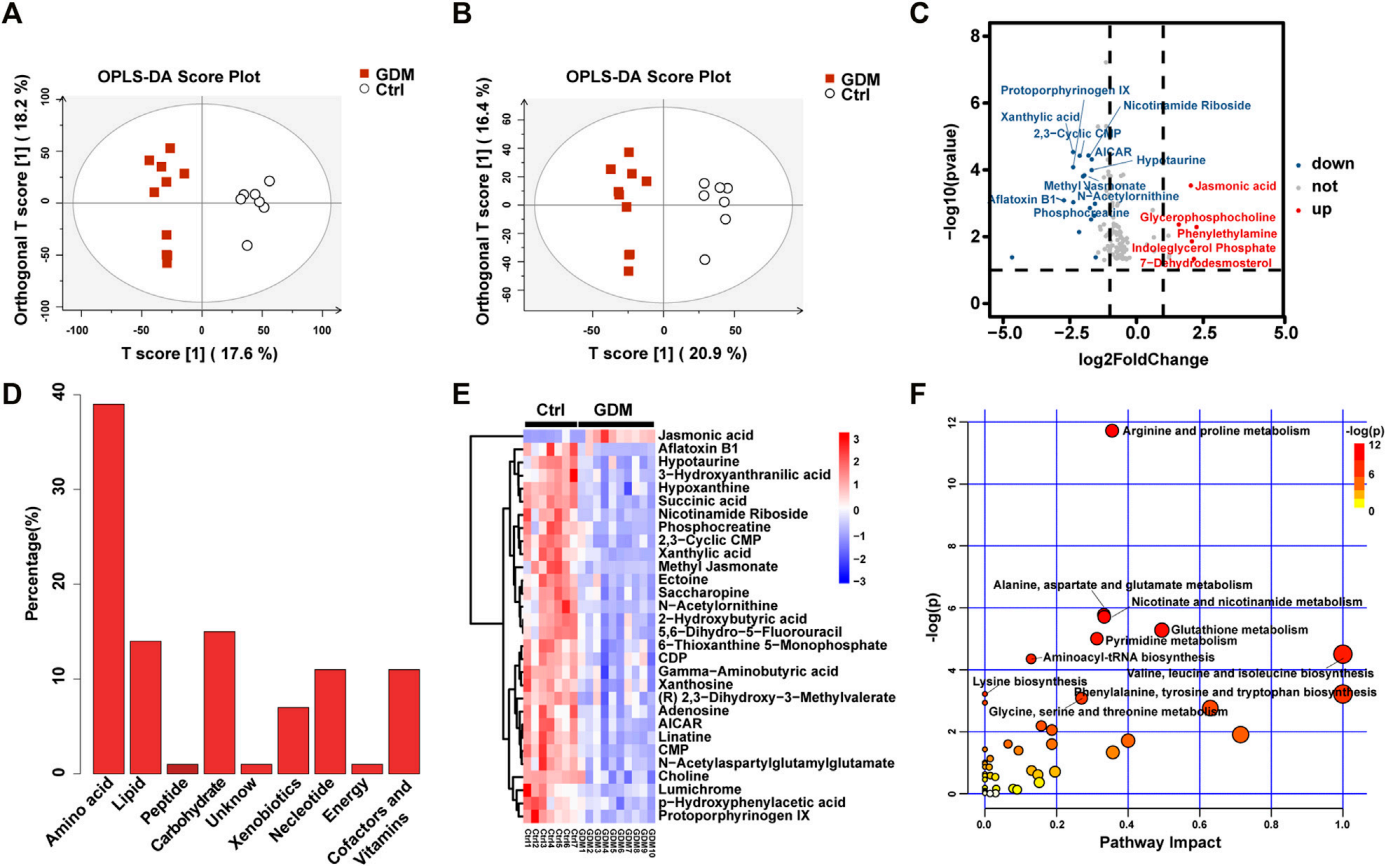
Metabolite analysis of the GDM and control groups. **(A)** Orthogonal partial least squares discriminant analysis (OPLS-DA) in positive and **(B)** negative ion mode was utilized to display the variance of the samples. **(C)** The volcano indicates the changes in fetal brain metabolite profiles. Metabolites up-regulated or down-regulated by |log_2_FoldChange| > 1.5 are shown in red and blue, respectively. **(D)** Bar plot of classification of the detected metabolites into major functional classes. **(E)** Heatmap presentation of the top 30 differential metabolites in fetal brains. **(F)** Different metabolic pathways in GDM fetal brains. Each bubble represents a metabolic pathway. The bubble size is related to the impact of the pathway and color (varying from yellow to red) means the metabolites are in the data with different levels of significance.

The UPLC-MS-based metabolic profiling method was performed to explore the potential biochemicals involved in the epigenetic regulation of the GDM group. The GDM group could be distinguished from the Ctrl group in the OPLS-DA score plot in both positive and negative ionization modes, which suggested that intrauterine hyperglycemia could induce distinct changes in fetal brain metabolites (Figures 5A,B).

**FIGURE 6:**
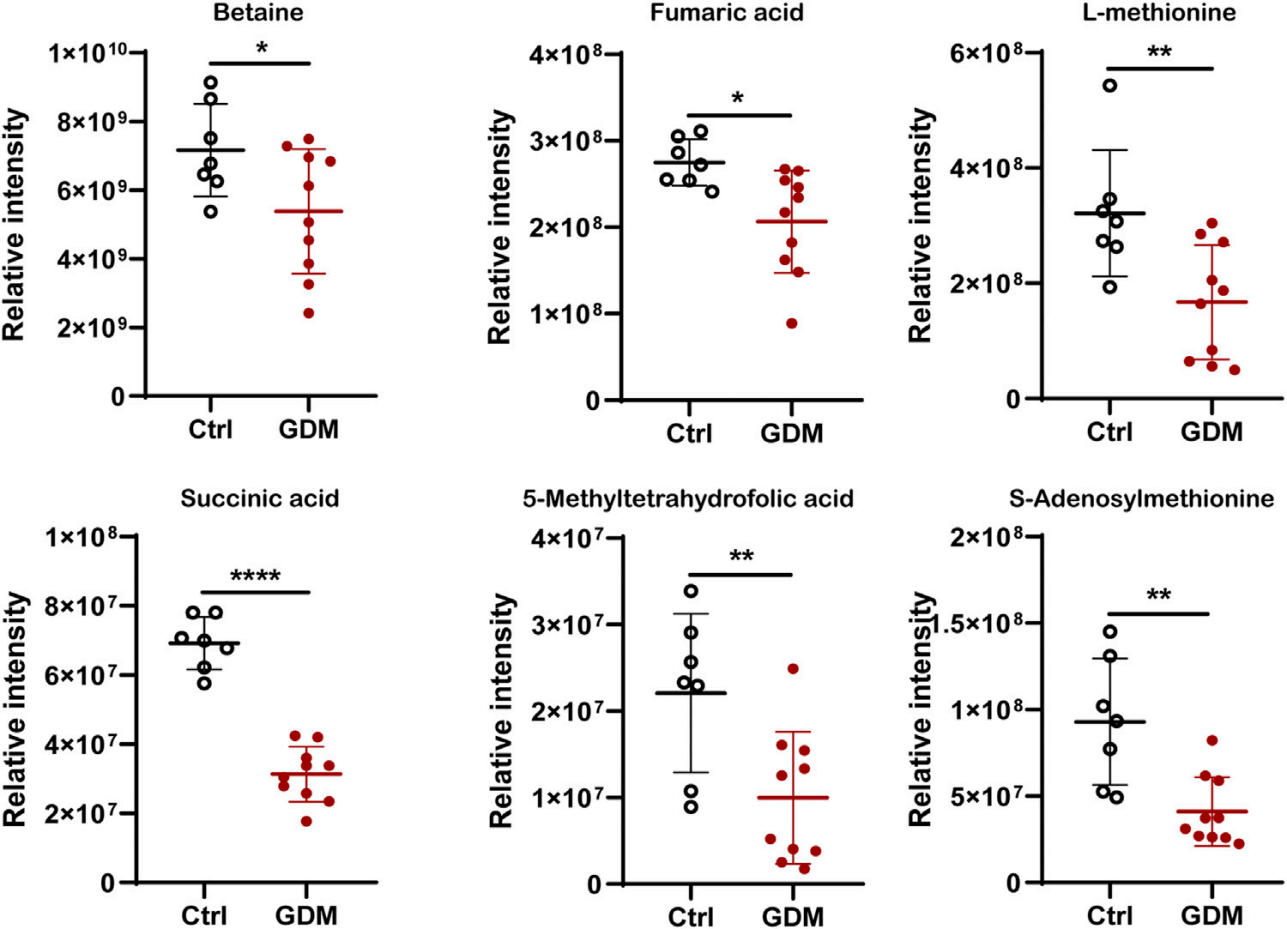
Comparison of the intensity of representative metabolites related to epigenetic modification between the control and GDM groups. *p < .05 vs. Control; **p < .01 vs. Control; ** **p < .0001 vs. Control.

In total, 123 significantly differential metabolites were identified in fetal brain tissues using the threshold of *p* < .05 and VIP ≥ 1, of which 8 up-regulated and 115 down-regulated in GDM group (Supplementary Table S4). Metabolites determined under the condition of |log_2_FoldChange| > 1.5 are shown in Figure 5C. Differential metabolites were in various classes, including “amino acid” (39%), “nucleotide” (11%), “carbohydrate” (15%), “lipid” (14%), “cofactors and vitamins” (11%), “xenobiotics” (7%), “peptide” (1%) and “energy” (1%) (Figure 5D). The top 30 metabolites included nicotinamide riboside, AICAR, gamma-aminobutyric acid, 2-hydroxybutyric acid, methyl jasmonate, choline, 3-hydroxyanthranilic acid and adenosine, most of which were related to cognition (Figure 5E). Further target metabolic pathways were screened out according to enrichment analysis (*p* value < .05) and pathway topological analysis (impact value >0.10). A total of 51 metabolic pathways were altered in response to intrauterine hyperglycemia, including “cysteine and methionine metabolism,” “citrate cycle (TCA cycle),” “pantothenate and CoA biosynthesis,” and “one carbon pool by folate,” (Figure 5F, Supplementary Table S5). In the GDM fetal brains, metabolites related to epigenetic modification were significantly down-regulated, including betaine, fumaric acid, L-methionine, succinic acid, 5-methyltetrahydrofolic acid, and S-adenosylmethionine (SAM) (Figure 6).

### Interactome Network Analysis of the Transcriptome and Metabolome

**FIGURE 7:**
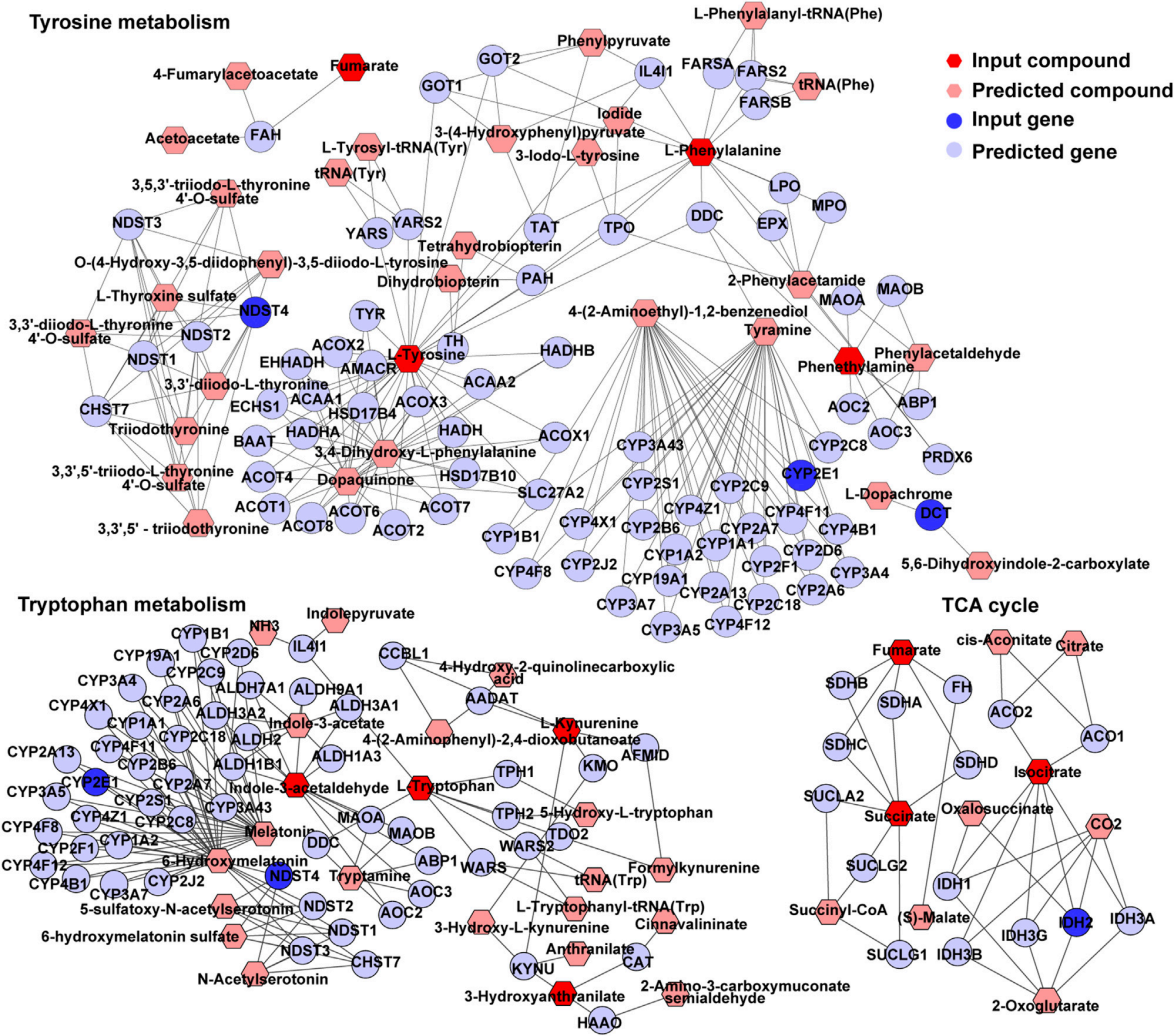
TCA cycle, tryptophan metabolism, and tyrosine metabolism in visual representation of the interaction between the transcriptome and metabolome. Dark blue circles represent differentially expressed genes; light blue circles represent inferred gene; dark red hexagons represent significantly changed metabolites; light red hexagons represent inferred metabolite; gray lines represent protein-protein or protein-metabolite interactions.

An interactome network model integrating the transcriptome and metabolome was generated, which connected pathways via gene-metabolite interactions. Analysis of this interactome network demonstrated that several critical metabolic processes were altered in the brains of GDM fetal mice, including “TCA cycle,” “tryptophan metabolism,” “tyrosine metabolism” (Figure 7). Other pathways included “purine metabolism,” and “urea cycle” and “metabolism of arginine, proline, glutamate, aspartate and asparagine” (Supplementary Figures S4, S5). The DEGs and differential metabolites associated with these pathways are presented in Table 1. Predicted genes were mostly belong to gene families such as cytochromeP450 family, which was likely to play roles in “tryptophan metabolism” and “tyrosine metabolism.” Predicted compounds such as citrate, succinyl-CoA in TCA cycle may interact with input compound and genes and co-mediate cognition disorder in GDM offspring. The results suggested that amino acid metabolism and TCA cycle may be the key processes associated with cognition of GDM offspring.

**TABLE 1:** Metabolic pathways identified in the interactome network, incorporating gene transcription and metabolite profiles.

Metabolic pathways enriched within the interactome network	Differentially expressed genes	Differential metabolites
TCA cycle	*Idh2*	Fumarate, Isocitrate, Succinate
Tryptophan metabolism	*Cyp2e1, Ndst4*	L-kynurenine, l-tryptophan, Indole-3-acetaldehyde, 3-Hydroxyanthranilate
Tyrosine metabolism	*Ndst4, Cyp2e1, Dct*	Fumarate, Phenethylamine, L-phenylalanine, L-Tyrosine
Purine metabolism	*Rev3l, Pole, Gda, Atp1b1, Atp2b2, Atp1a3, Kif3b, Nsf, Dnm3, Dnm1, Aday8, Eef1a2, Pde1b, Pde4d, Rrm1*	Guanine, Hypoxanthine, Xanthine, cAMP, L-aspartate, Fumarate, 5′-XMP, carboxamide, 1-(5′-phosphoribosyl)-5-amino-4-imidazole-, Xanthosine, Adenosine
Metabolism of arginine, proline, glutamate, aspartate and asparagine	*Asns, Nags, Gad1, Odc1*	L-proline, L-arginine, Glutathione, GSSG, SAM, L-aspartate, N-(L-arginino)succinate, Fumarate, spermine, Spermidine, L-lysine, 4-aminobutanoate, N-acetylornithine

## Discussion

The cognitive deficits of GDM offspring have been investigated for years in both epidemiological and experimental studies (Huerta-Cervantes et al., 2020; De Sousa, 2021). To explore the potential molecular mechanism, we are the first to profile the methylome and transcriptome simultaneously in the hippocampi of fetal GDM mice. Genomic and bioinformatic analysis showed that in the GDM group, differentially methylated and expressed genes were enriched in neural signal transmission of the synapse and cognitive function. We further performed a metabolome analysis of GDM fetal mice brains and found differential metabolites involved in one-carbon metabolism (including SAM, betaine, L-methionine and 5-methyltetrahydrofolic acid) and the TCA cycle (including succinic acid and fumaric acid) that affect DNA methylation modification.

The hippocampus has long been considered serving a critical function in cognition. Diabetes-induced cognitive impairment shows the ultrastructural of hippocampus (Liu et al., 2020). Neuronal degeneration and nucleus shrinkage in the hippocampus were also found in mice with d-galactose-induced cognitive dysfunction (Tu et al., 2018). Our ultrastructural data of hippocampi in fetal mice indicated irregular nucleus shapes and obvious nucleus shrinkage, which may be related to hippocampal deficits, indicating neurodegeneration in the central nervous system of GDM fetuses. This change may be one of the explanations of behavioral dysregulation in GDM offspring in previous studies.

It has been shown that maternal diabetes induces oxidative stress and reactive oxygen species (ROS) accumulation, which in turn alter the DNA methylation status in the early embryo (Li et al., 2005; Giacco and Brownlee, 2010). However, changes in DNA methylation in the fetal hippocampus of GDM have not been reported. In our study, the enrichment analysis of DMGs suggested changes in synapse signal transmission in the hippocampi of the GDM group. Methylome profiling revealed that the top DMGs were mostly involved in cognition and synapse changes. Previous study showed down-methylation of *Irs1* in patients with cognition disorder; *Sox5*, as a transcription factor, is a critical modulator of neurite outgrowth (Naudet et al., 2018); *Sorcs3* controls the proper positioning and mobility of glutamate receptors in the postsynaptic density, which is crucial to synaptic function and plasticity (Christiansen et al., 2017); and *Specc1* has a strong link to cognitive abilities by remodeling small GTPases in the cytoskeleton (Maroteaux et al., 2012). *Ash1L* is a kind of histone methyltransferase. The mutation or loss of *Ash1L* may cause autistic-like behaviors (Satterstrom et al., 2020; Gao et al., 2021); and low expression of *Serpinf2* is related to Alzheimer’s disease (De Jager et al., 2014). *Fendrr* has been proven to be significantly increased in the brains of diabetes patients (Wang et al., 2021). The development of the nervous system is under the strict control of a number of signal transduction pathways, which are often interconnected. The enrichment analysis of DMGs suggested changes in synapse signal transmission in the hippocampi of the GDM group. Previous studies revealed that dendrite development and its plasticity regulation subserve cognitive functions in the brain (Forrest et al., 2018).

The top DEGs have particular roles in cognition and neurodevelopment. *Nrgn* can modulate synaptic plasticity by encoding neurogranin, and a decrease in neurogranin leads to cognitive deficiency (Hwang et al., 2021). Knockdown of *Hap1* promotes specification of supernumerary axons in primary hippocampal neurons and promotes disorders of cognition (Mejia et al., 2013). *Hap1* was enriched in the “GABAergic synapse” in the KEGG pathway of DEGs. The reduced expression of *Slc12a5* plays a key role in neurodevelopmental delay (Hinz et al., 2019). *Trim9* deletion alters the morphogenesis of developing and hippocampal neurons and impairs spatial learning and memory (Winkle et al., 2016). In our data, those genes displayed an identical down-regulated trend. The function of these DEGs includes regulation of synapse organization and neurotransmitter transport, which is consistent with the result of hippocampal transcriptome in adult offspring of GDM in our previous study (Zou et al., 2021). This suggests that the effects of intrauterine hyperglycemia on the fetal hippocampus will continue into adulthood.

By overlapping the sequencing results of the methylome and transcriptome, we identified genes such as *Shank2*, *Ank1*, *Sez6l* and *Sema5b*, which were differentially methylated and expressed in the fetal hippocampus of GDM as factors that may contribute to the cognitive deficits caused by intrauterine hyperglycemia. *Shank2* was hypermethylated and down-regulated. A previous study showed that mutant mice carrying *Shank2* deletions exhibited behavioral defects (Monteiro and Feng, 2017); overexpression of full-length *Sema5b* in hippocampal neurons reduced synapse number (O’Connor et al., 2009). Functionally connected predicted interactor genes were all down-regulated in transcriptome profiling, including 3 hypermethylated genes (*Pcdha6*, *Ank1*, *Sez6l*) and 4 hypomethylated genes (*Kcnb1*, *Kcnj6*, *Prkcb*, *Sorcs3*). *Pcdha6* showed decreased expression in psychiatric illness (Srikanth et al., 2018). Methylome profiling implicates hypermethylation of *Ank1* in Alzheimer’s disease (Lunnon et al., 2014). *Sez6* has been shown to influence synapse numbers and dendritic morphology, and *Sez6* TKO mice have impaired motor learning and negatively affected motor coordination (Nash et al., 2020; Qiu et al., 2021).

These changes highlight the importance of epigenetic regulation in environmentally induced diseases affecting neurocognition. DNA methylation refers to the transfer of the methyl group provided by SAM to the 5-position carbon atom of cytosine (Su et al., 2016). In GDM fetal brains, metabolomic analysis data showed decreased levels of SAM, indicating the mechanism of DNA methylation alteration induced by intrauterine hyperglycemia. Emerging studies have shown that metabolites can regulate DNA methylase activity. Metabolomics identified six down-regulated metabolites, fumaric acid, betaine, L-methionine, succinic acid, 5-methyltetrahydrofolic acid, and SAM, which may influence the methyl donor pool. One-carbon metabolism links the maternal environment with epigenetic regulation and early development (James et al., 2018). Betaine functions primarily as a methyl donor substrate in one-carbon metabolism to convert homocysteine to methionine by betaine homocysteine methyltransferase (BHMT), which is critical for embryonic and fetal development. Methionine then converts to SAM, the universal methyl donor for DNA and protein methylation processes that are essential for epigenetic gene regulation (Zhao et al., 2017). 5-Methyltetrahydrofolate was used as a methyl donor to catalyze the remethylation of homocysteine to methionine (Froese et al., 2019). Additionally, succinate and fumarate can compete with α-ketoglutarate in the active site of TETs, inhibiting their function (Hitchler and Domann, 2012). Thus, decreased succinate and fumarate could also lead to hypomethylation of the DNA. Additionally, our present study just showed a greater number of genes with hypomethylation in the fetal hippocampus exposed to GDM, the global DNA methylation status should be validated by the other technologies in the future research.

In addition to affecting methylation status, some metabolites may directly induce cognitive dysfunction in GDM fetal offspring. Succinic acid decreased in mice with a deterioration of spatial learning and memory (Ning et al., 2018). Nicotinamide riboside was reported to be beneficial for hippocampal neurodevelopment (Ear et al., 2019). AICAR can improve cognition in young and aged mice (Kobilo et al., 2014). GABA, a necessary neurotransmitter, alters cognitive dysfunctions in depression and other brain disorders (Prévot and Sibille, 2021). Methyl jasmonate may be useful in conditions associated with memory dysfunctions or age-related cognitive decline (Eduviere et al., 2015). Choline is a micronutrient and a methyl donor that is required for normal brain growth and development and produces profound benefits on cognition (Velazquez et al., 2019).

The interactome network of the transcriptome and metabolomics showed changes in amino acid metabolism, TCA cycle and purine metabolism. Previous study showed pathways involved in the metabolism of amino acids and tricarboxylic acid cycle (TCA) were enriched in metabolites whose levels were higher in the neonatal brains (Chen et al., 2021). Amino acids are vital for proper neurodevelopment, as they comprise the most abundant neurotransmitters in the brain and act as neurotransmitter precursors and neurotrophic factors, which induce synaptogenesis and neuronal proliferation (Martínez García et al., 2018). Evidence has accumulated in the last decades pointing to a contribution of purines to brain development including embryonic neurogenesis, migration of principal neurons and interneurons, guidance for neuronal connectivity, synaptogenesis and synaptic stability/elimination (Rodrigues et al., 2019). Thus, the change of amino acid metabolism, TCA cycle and purine metabolism will be associated with dysfunction of brain development. This result built a latent relationship of differential metabolites and DEGs in the pathway and provided a new sight into the molecular mechanism of cognitive dysfunction in GDM, which deserve further attention in our future research.

Our study provides novel experimental evidence about the adverse effects of intrauterine hyperglycemia of GDM on fetal hippocampus, combined with our previous study of cognition in GDM adult offspring, revealing that the fetal brain is very sensitive to intrauterine metabolic disorders. Although our findings were generated in mouse model, it is important to recognize that if exposed to maternal GDM environment, the long-term health follow-ups and lifestyle interventions are necessary to the offspring. Meanwhile, the findings indicate that the most important and effective way to reduce the risk of offspring is to detect and intervene metabolic abnormalities in earlier pregnancy period, and eliminate the risk factors of gestational diabetes. The main limitations of this study are the small sample size. Despite the consistency within the group, which is probably enough to garner some key information, in the future research more sample are needed to validate the findings. The potential mechanism between metabolites and epigenetic regulation is worth further study.

## Conclusion

Our findings suggest the underlying mechanism for the developmental change of fetal hippocampus exposed to maternal gestational diabetes. GDM exposure induced hippocampal nucleus shrinkage in the CA1 area and changed gene methylation and expression of the hippocampus, as well as metabolites in the brain. Mechanistically, our data provide novel evidence that intrauterine hyperglycemia exposure alters methylation status, which may be affected by brain metabolism, and leads to gene changes contributing to cognitive disorders in GDM offspring.

## Data Availability

Publicly available datasets were analyzed in this study. This data can be found here: Metabolomics: www.ebi.ac.uk/metabolights/MTBLS3181 RRBS: https://www.ncbi.nlm.nih.gov/sra/PRJNA731602 RNA-seq: https://www.ncbi.nlm.nih.gov/sra/PRJNA731601.

## Supplementary Material

Supplementary Figure S1 Experimental study design. **(A)** A hyperglycemic environment lasted throughout pregnancy, and the hippocampus was isolated from the fetus at embryonic day 18.5. **(B)** A hyperglycemic environment appeared in the middle and late trimesters of most clinical GDM cases, and brains were isolated from the fetus at embryonic day 18.5. **(C)** Maternal glucose levels at day 3, day 7 and day 18.5 of pregnancy in the first model. **(D)** Cross-talk of the methylome and transcriptome from the hippocampus and metabolomics from the brain.

Supplementary Figure S2 Supervised PCA of DMLs revealed a clear distinction between GDM and control samples.

Supplementary Figure S3 Kyoto Encyclopedia of Genes and Genomes (KEGG) enrichment analysis based on shared genes of RNA-seq and RRBS. The color gradient from dark purple to dark pink in the heatmap indicates fold change of DEGs. The bubble size is related to the impact of the pathway.

Supplementary Figure S4 Purine metabolism in visual representation of the interaction between the transcriptome and metabolome.

Supplementary Figure S5 Urea cycle and metabolism of arginine, proline, glutamate, aspartate and asparagine in visual representation of the interaction between the transcriptome and metabolome.
