# Supplementary figures and images for: Integrated Multi-Omics Analysis Reveals the Effect of Maternal Gestational Diabetes on Fetal Mouse Hippocampi
Source: Front Cell Dev Biol. 2022 Feb 14;10:748862. doi: 10.3389/fcell.2022.748862 (PMC8883435; doi:10.3389/fcell.2022.748862)

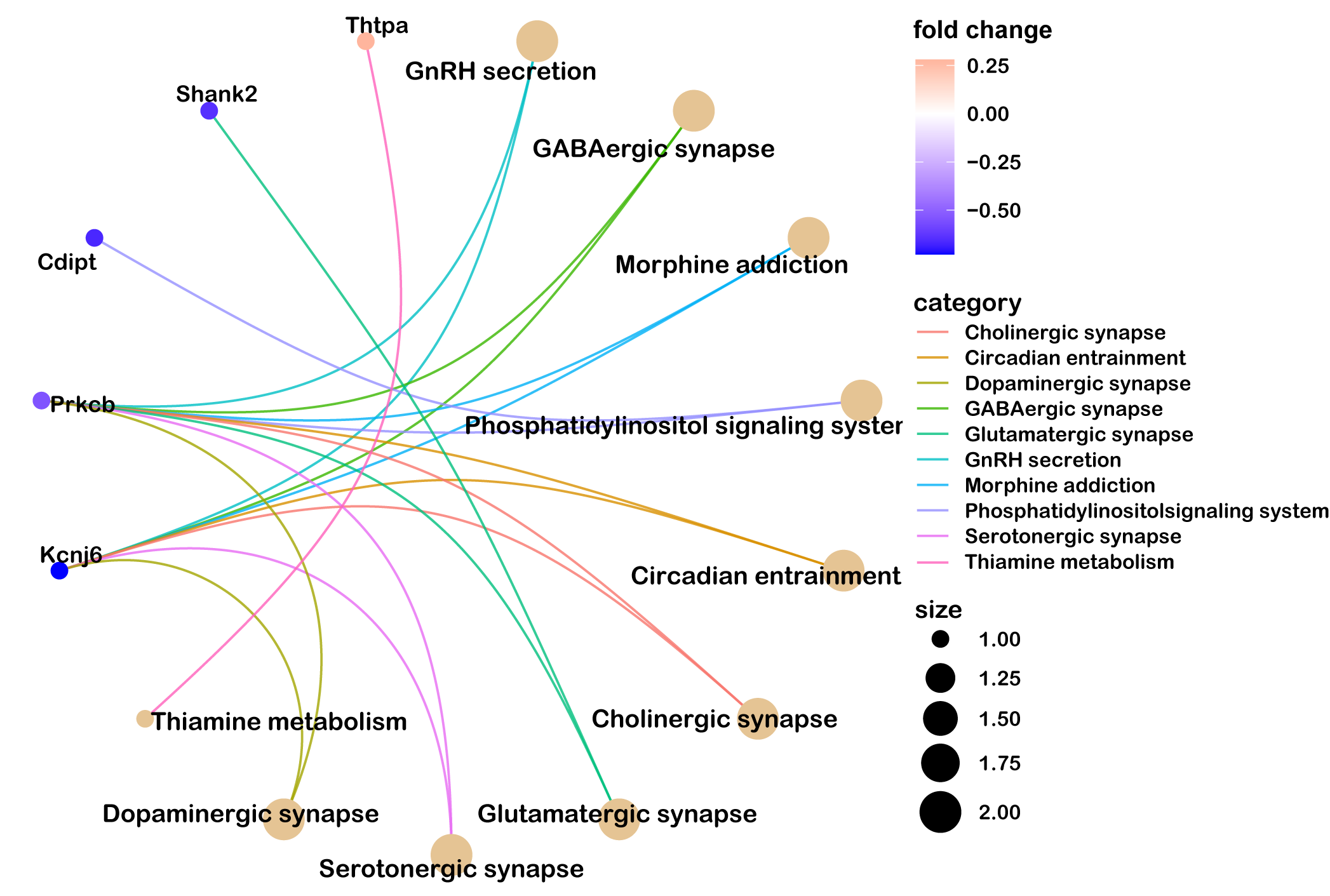

Supplement: Supplementary file 3 [file Image3.tif]

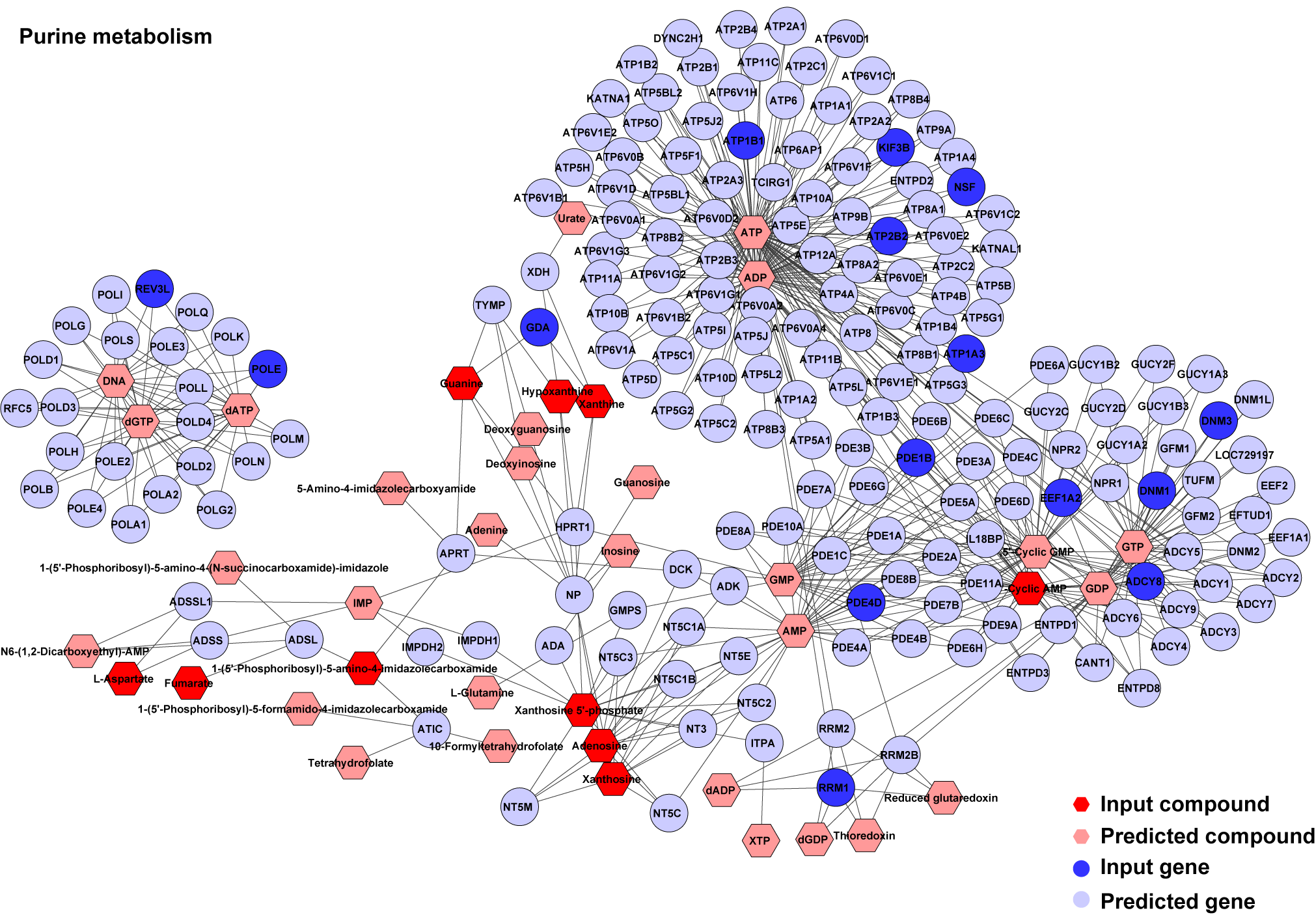

Supplement: Supplementary file 4 [file Image4.tif]

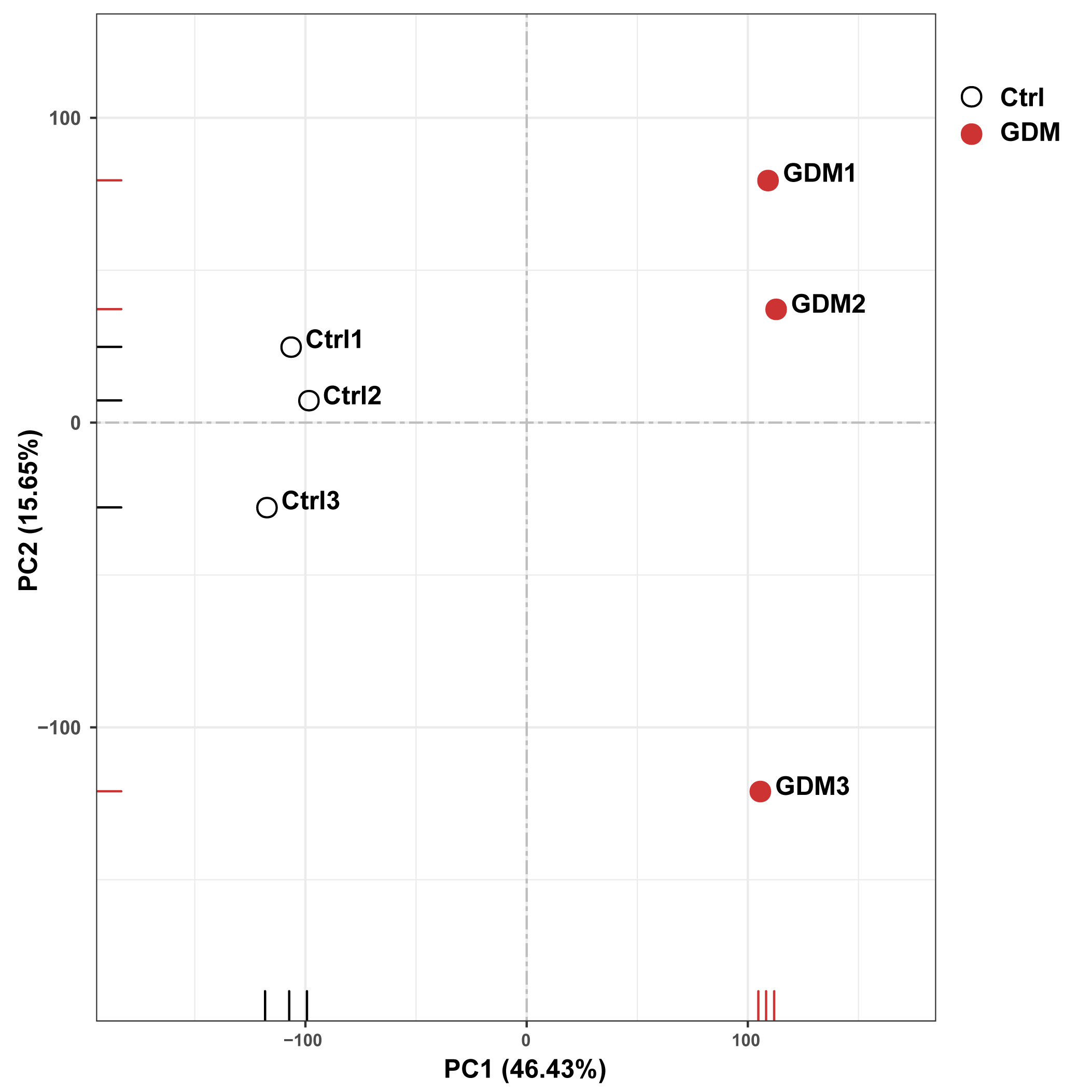

Supplement: Supplementary file 5 [file Image2.tif]

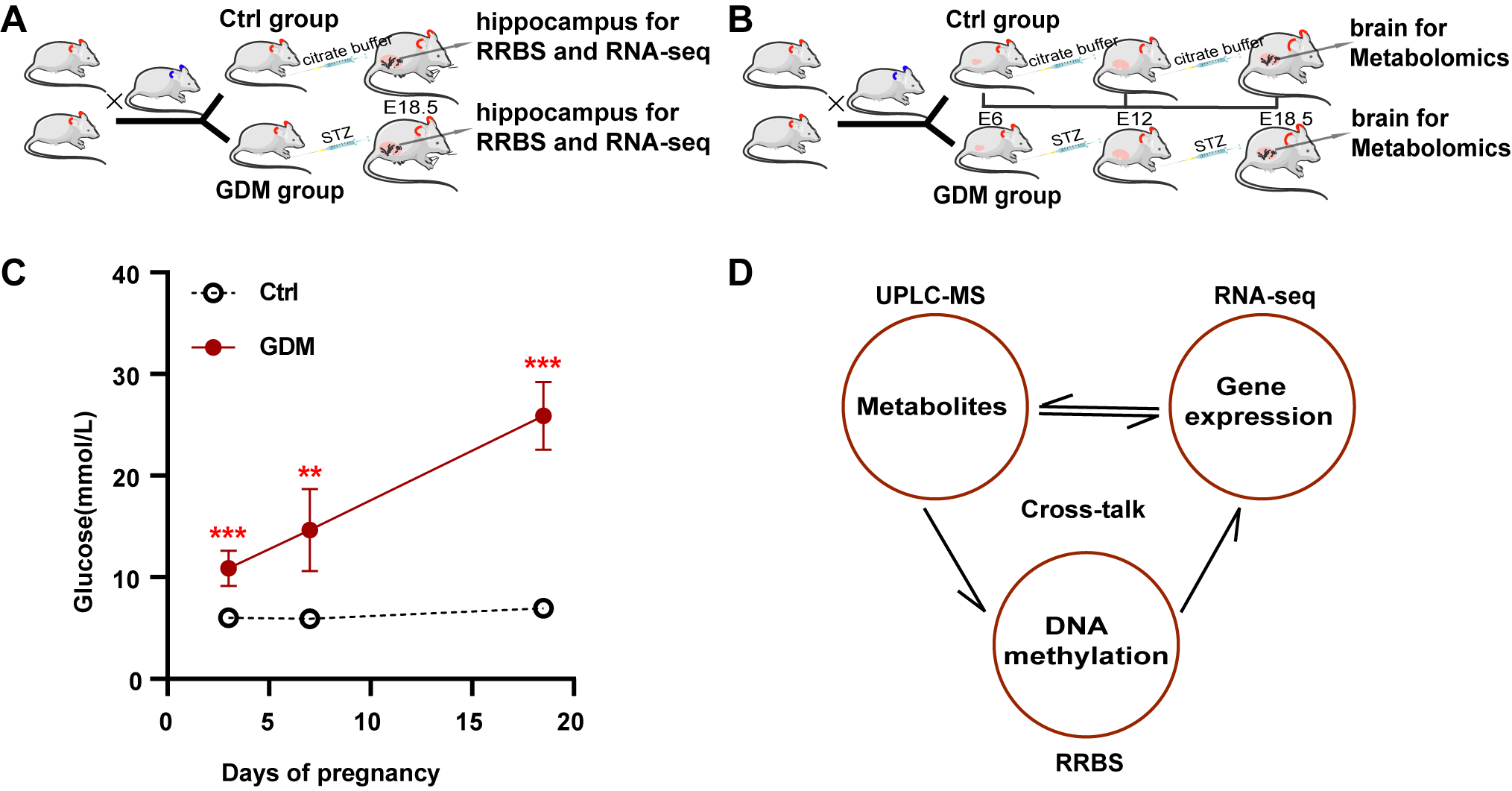

Supplement: Supplementary file 6 [file Image1.tif]

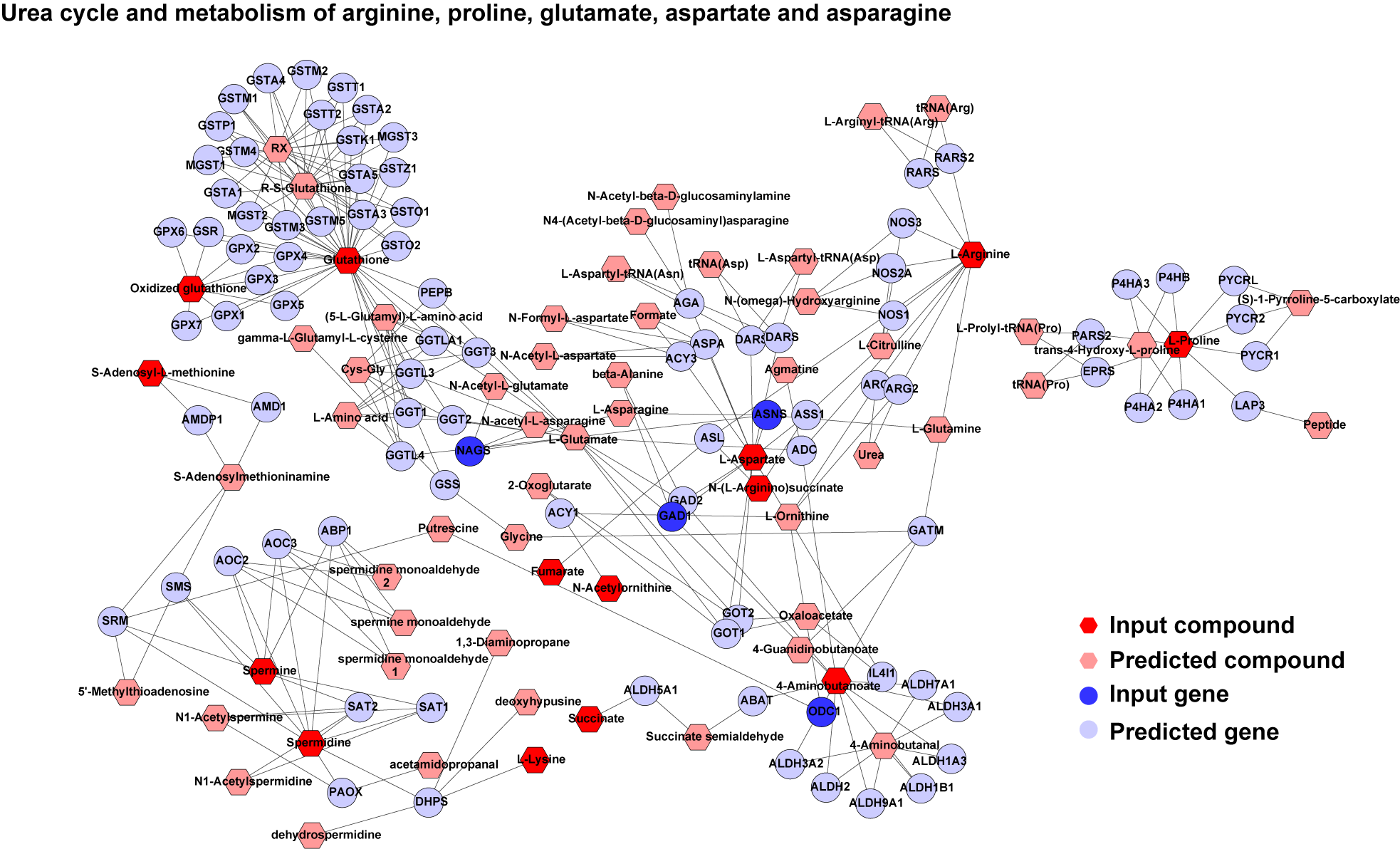

Supplement: Supplementary file 9 [file Image5.tif]
